# Efficacy of electrical stimulation on epidural anesthesia for cesarean section: a randomized controlled trial

**DOI:** 10.1186/s12871-020-01063-1

**Published:** 2020-06-10

**Authors:** Young Sung Kim, Hyo Sung Kim, Hyerim Jeong, Chung Hun Lee, Mi Kyoung Lee, Sang Sik Choi

**Affiliations:** Department of Anesthesiology and Pain Medicine, Korea University Guro Hospital, 148 Gurodong-ro, Guro-gu, Seoul, 08308 South Korea

**Keywords:** Cesarean section, Electrical stimulation, Epidural anesthesia

## Abstract

**Background:**

Loss of resistance (LOR) technique is a widely used method to identify the epidural space. However, cases of inadequate epidural anesthesia in cesarean section were frequently reported. Also, the success rate of epidural anesthesia with LOR technique varied depending on the proficiency of the practitioner. The purpose of this study was to assess the efficacy and safety of electrical stimulation to identify epidural spaces in cesarean section for novices or clinicians with recent gap in experience.

**Methods:**

Pregnant women scheduled for elective cesarean section were randomly allocated to two groups. Groups were classified based on the methods used for identifying the epidural space: the LOR group (group L) and the LOR with epidural electrical stimulation group (group E). Clinicians with less than 10 epidural cesarean section experiences in the recent year performed epidural anesthesia for cesarean section. In the group E, a RegionalStim® conductive catheter was inserted through the Tuohy needle, and the guidewire passing through the catheter was connected to a peripheral nerve stimulator. The intensity of the stimulation was gradually increased from 0.25 mA to 1.5 mA until paresthesia was elicited and radiated. We assessed the success of epidural anesthesia (complete success, partial success or failure). Other clinical parameters including maternal satisfaction, time required for epidural anesthesia, neonatal Apgar scores, pain scores and adverse events were compared between the two groups.

**Results:**

Except for 6 patients who withdrew consent, 54 patients were enrolled in this study (28 for the group L and 26 for the group E). The demographic data showed no difference between the two groups. There was no adverse event resulted from electrical stimulation. The group E showed higher rate of complete success, sensitivity in finding epidural space and maternal satisfaction compared to the group L (21/26 vs. 15/28, *p* = 0.034, 0.96 vs. 0.68, *p* = 0.012 and 4.04 vs. 3.39, *p* = 0.02, respectively). The other clinical parameters showed no differences between the two groups.

**Conclusion:**

In addition to the conventional LOR technique, identifying epidural spaces using electrical stimulation led to better outcomes without additional risks for novices as well as clinicians with recent gap in experience.

**Trial registration:**

This study was retrospectively registered in the ClinicalTrials.gov Registry (NCT03443466) on February 23, 2018.

## Background

Epidural analgesia and anesthesia are widely used in the obstetrical area. Especially in labor, the usefulness of epidural analgesia is remarkable [1]. Epidural analgesia showed high maternal satisfaction as mothers can walk and move freely with effective labor pain relief [2]. In the cases for cesarean section, epidural anesthesia also demonstrated many advantages than other anesthetic techniques especially for the parturient with congenital heart disease [3]. However, spinal anesthesia appeared to be preferred over epidural anesthesia in the cesarean section [4]. Compared to the epidural analgesia, epidural anesthesia for cesarean section requires more intense anesthesia with wider dermatome levels. When converting epidural labor analgesia into surgical anesthesia for cesarean section, the rate of conversion failure has been reported up to almost 20% [5]. Interestingly, the rate of failed epidural anesthesia conversion was 4.5 times higher for non-specialist anesthesiologists than among obstetric anesthesiologists [6].

In clinical practices, the success rate of epidural anesthesia varied depending on the proficiency of the practitioner [7]. Moreover, from a teaching standpoint, it was difficult to determine immediately whether a resident or novice practitioner had properly performed epidural anesthesia. In the case of spinal anesthesia, the success of the procedure can be predicted mostly by free CSF flow. On the other hand, loss of resistance (LOR) technique, which is widely used for confirmation in epidural anesthesia, is relatively subjective to interpret. Kopacz. et al. [8] reported 60 attempts at epidural anesthesia may be necessary to achieve a 90% success rate. We expected that untrained practitioner would have problems, especially in finding epidural spaces. In addition, proper placement of the epidural catheter may be difficult, particularly when performed by a novice provider.

We have previously shown the utility of electrical stimulation-guided epidural analgesia for vaginal delivery [9]. We thought that electrical stimulation would be also applied to the epidural confirmation for cesarean section. In the present study, we demonstrated the efficacy and the safety of electrical stimulation when confirming epidural spaces in cesarean section. We hypothesized that electrical stimulation-guided epidural anesthesia may help to find a correct epidural space and may result in a higher success rate for novices or clinicians with recent gap in experience.

## Methods

### Study population

This study was a single-center prospective randomized controlled trial conducted at Korea University Guro Hospital from 2018 to 2019. After obtaining approval from the Korea University Guro Hospital Institutional Review Board (IRB number 2015GR0703), written informed consent was obtained from all subjects participating in the trial. The trial was retrospectively registered at the ClinicalTrials.gov (trial identifier: NCT03443466). The current study was presented in accordance with the Consolidated Standards of Reporting Trials (CONSORT) guidelines. All patients were recruited from the Department of Obstetrics and Gynecology, Korea University Guro Hospital, by the research staff and were enrolled in the study at the hospital before surgery. Written informed consent was obtained from all participants after providing an explanation of the trial.

Patients aged 20 to 42 years, at 36 to 41 weeks’ gestation, of American Society of Anesthesiologists (ASA) physical status I to II, and scheduled for cesarean section under epidural anesthesia were included in the study. Patients with severe cognitive impairment, skin infection on the back, history of lidocaine allergy and lumbar surgery, lumbar deformity, and hemostatic disorder were excluded from the study. Those undergoing anticoagulant therapy, having cardiopulmonary compromised status and expecting twin delivery, as well as those who refused to participate were also excluded. Demographic data including age, weight, height, and ASA class were collected from all the patients (Table 1). Patients were randomly allocated to the LOR group (group L) or the LOR + electrical stimulation group (group E), and they were unaware of the group assignment before the surgery. A single independent investigator was responsible for the random allocation sequence generation and the group assignment of patients. Randomization was achieved into 2 blocks using a web-based computer-generated list (www.randomization.com). The subject numbers were kept in opaque, sealed envelopes that were opened in the operating room by an independent anesthesiologist not involved in the study. Both the patients and principal investigator were blinded to group allocation.

**Table 1:** Demographic data

	Group L (***n*** = 28)	Group E (***n*** = 26)
Age (years)	35.93 ± 2.54	35.15 ± 4.76
Weight (kg)	66.56 ± 8.74	71.18 ± 6.69
Height (cm)	159.48 ± 6.10	160.65 ± 7.67
Body mass index (kg/m^2^)	26.17 ± 3.09	27.67 ± 4.00
ASA class (I / II)	7 / 21 (25 / 75)	6 / 20 (23 / 77)
Status of practitioners (R1 / R2 / R3 / R4 / Specialist)	4 / 8 / 2 / 6 / 8	4 / 8 / 2 / 7 / 5

Values are either the mean ± SD or the number of patients (%). Group L used a loss of resistance technique in the epidural cesarean section while group E used electrical epidural stimulation combined with a loss of resistance technique. There was no significant difference between the two groups. ASA class refer to American Society of Anesthesiologists physical status. R1, R2, R3 and R4 refer to first-, second-, third- and fourth-year residents

### Anesthetic protocol

Routine monitoring devices including non-invasive blood pressure measurement, electrocardiogram and pulse oximetry were applied to each patient. We also monitored temperature and urine output during the perioperative period. The baseline values for each measurement were recorded before anesthesia induction. Epidural procedures were performed by anesthesiologists who were novice or with a recent gap in experience (less than 10 experiences for epidural cesarean section in the recent year). A resident who meets the conditions first attempted the procedure. If the residents did not find an epidural space after two attempts, another anesthesiologist who was a specialist performed the procedure. We considered that the practitioners have used one chance to attempt in any of the following circumstances: a) he or she gave up finding an epidural space and changed the injection site, b) the epidural catheter could not be inserted, and c) the leakage of blood or CSF was suspected. They did not know the group assignment for each patient until the test dose administration. The level of their anesthetic experience and status, ranging from first year to fourth year (resident or specialist), were recorded for each case.

After oxygen mask application with 5 L/min of oxygen, for the epidural catheter placement, patients were placed in the left lateral decubitus position. Once a sterile drape was performed, a local infiltration with 1% lidocaine was done at the L2/3 intervertebral level. A midline approach was used with a 17-guage Tuohy needle. Epidural space was then identified using LOR with air. After ensuring no cerebrospinal fluid or blood, 3 ml of 1% lidocaine (30 mg) was administrated as a test dose. In the group E, an epidural catheter (20-gauge, open tip catheter, 800 mm of length, Regional Stim®, Sewoon Medical Co., Ltd., Korea) with a conductive guidewire (Nitinol, 1100 mm of length; 800 mm inside the catheter and 300 mm exposed for connection to the electric nerve stimulator) was inserted 3 cm cephalad beyond the tip of the Tuohy needle right after a test dose administration. The cathode of the electric nerve stimulator (Stimuplex® HNS 12, B. Braun Melsungen, Germany) was connected to the exposed guidewire, and the anode was attached to an electrode on the patient’s calf. For stimulation, the electric current was gradually increased from 0 mA to 2 mA, with a frequency of 1 Hz and pulse-width of 300 ms. A minimum required current to elicit paresthesia of a dermatome or motor response of a muscle group including the hip adductors, iliopsoas, gluteus, and hamstrings was recorded for each patient. Usually the current was checked within 3 min, so once epidural space was confirmed by electrical stimulation, the guidewire was removed, and the patient was observed for the remaining time until 3 min past the test dose administration. If there was no adverse sign and intrathecal injection was not suspected, 18 ml of 2% lidocaine was injected via the epidural catheter as a main dose. In the group L, the same process followed the test dose administration except the electrical stimulation. The same guidewire embedded epidural catheter was inserted, and the guidewire was immediately removed. 3 min after the test dose administered, 18 ml of 2% lidocaine was injected via the epidural catheter as a main dose. The patient was thereafter placed on the supine position.

The cold sensory test was performed with an alcohol swab. The additional epidural bolus, 5 ml of 0.75% ropivacaine, was injected through the epidural catheter when the cold sensory blockade did not reach a T7 level in 10 min after the main dose administration or when the patient complained of pain during surgery. After the baby was delivered, 2 ample (20 IU) of oxytocin was mixed to the main fluid for the mother, and the Apgar scores of the baby were assessed at 1 min and 5 min. When hypotension occurred during the perioperative period, ephedrine 4 mg or phenylephrine 50mcg was administered intravenously to keep the systolic blood pressure at − 20 to 20% of the baseline value. Any complications were recorded during the perioperative period.

### Study endpoints

The primary outcome was a success rate of epidural anesthesia. We assessed each case as “complete success”, “partial success” or “failure”. If there were no signs for motor and sensory blockade in 10 min after epidural main dose administration, we regarded it as a “failure” and the anesthesia was converted to general anesthesia. If epidural anesthesia did not reach appropriate range and intensity and if the additional ropivacaine bolus was required, it was regarded as a “partial success”. If the anesthesia was maintained enough to proceed with surgery, it was considered as a “complete success”. The rate of complete success in epidural anesthesia was calculated as “complete success / complete success + partial success + failure”. And the sensitivity in finding the epidural space was calculated as “complete success + partial success / complete success + partial success + failure” in each group. Meanwhile, calculation of the specificity was not available due to methodological limitation.

The secondary outcomes include Apgar scores, presence or absence of hypotension and ephedrine or phenylephrine use during perioperative periods, anesthetic times, fluids, transfusion, urine output, blood loss, pain score 1 h after arrival at PACU, maternal satisfaction, nausea/vomiting and other complications. One- and 5-min Apgar scores were compared to assess the effects of epidural electrical stimulation on the neonate. Anesthesia time was recorded in detail as described below: Time interval between operation room admission and the start of the operation, time A (interval between drape and the test dose administration), time B (time it took to confirm epidural space, place epidural catheter and administer main dose), operation time, time interval between the end of the operation and discharge to the post-anesthesia care unit (PACU). A blinded independent anesthesiologist assessed postoperative pain and other complications in the PACU. Pain was assessed by a visual analogue scale (VAS) score on an 11-point scale, where 0 indicates no pain and 10 refers to unbearable pain. Maternal satisfaction was evaluated by a postpartum interview. It was rated on a scale ranging from 1 to 5, where 1 represents very unsatisfied and 5 represents very satisfied.

### Sample size and statistical analysis

A power analysis suggested that a minimum sample size of 26 patients for each group would be required with a significance level of 5% to achieve a power of 90%. It was calculated from our preliminary data: complete success rates of 0.9 for electrical stimulation group and 0.5 for conventional group. To allow for an exclusion rate, the study population was prospectively set at 60 patients.

Statistical analyses were performed with SPSS 22 (IBM, Armonk, NY, USA, Statistical Package for the Social Science 22). The outcomes were assessed based on the intention-to-treat analysis. Data expressed as mean ± standard deviation were tested for normality using the Kolmogorov-Smirnov test and were compared using independent t-tests or Mann-Whitney U tests depending upon the results of the Kolmogorov-Smirnov analysis. Finally, data expressed as the number of patients were compared using chi-square analysis or Fisher’s exact test as appropriates. A *p*-value of < 0.05 was considered significant.

## Results

**Fig. 1:**
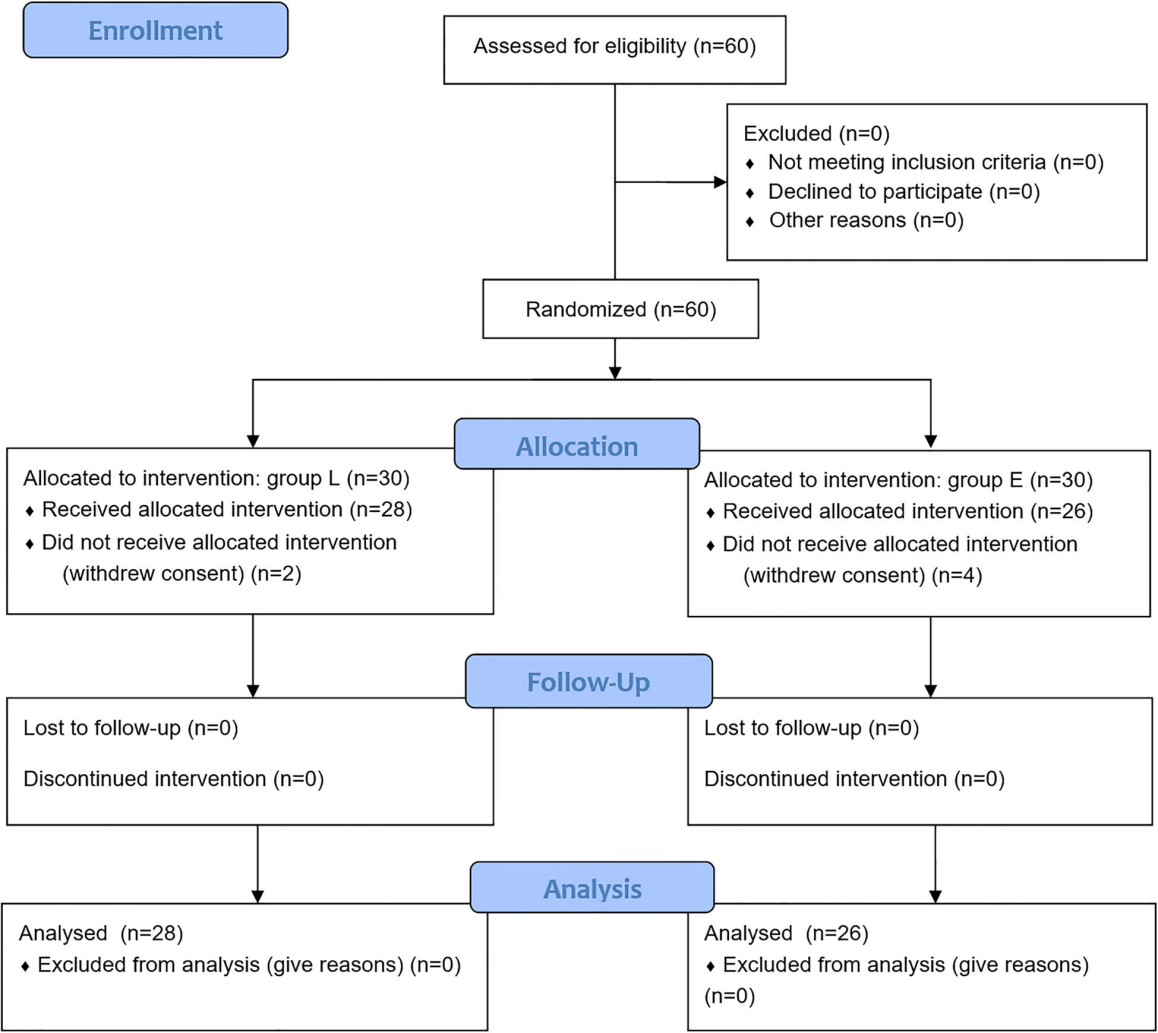
CONSORT flow diagram. Group L used a loss of resistance technique in the epidural cesarean section while group E used electrical epidural stimulation combined with a loss of resistance technique

The CONSORT flow diagram is presented in Fig. 1. Total 54 patients were enrolled in this study (28 patients in the group L and 26 patients in the group E) except 6 patients who withdrew consent due to anxiety and family opposition with no particular event prior to the cesarean section.

Demographic data including age, weight, height, body mass index, and ASA class showed no significant differences between the two groups (Table 1). The minimum electric current used to evoke paresthesia or muscle contraction in the group E was 1.06 ± 0.36 mA. Unilateral response was observed in 16 cases (62%) while bilateral response was observed in 10 cases (38%) (Table 2). Unilateral response did not correlate with “partial success” or “failure”. Patients with unilateral and bilateral responses exhibited comparable patterns, such that those with unilateral responses reported 13 “complete success” and 3 “partial success” and that those with bilateral responses reported 8 “complete success”, 1 “partial success” and 1 “failure”. (Table 2).

**Table 2:** The outcomes for the electrical stimulation

	*N* = 26
The minimum electric current (mA)used to evoke paresthesia or muscle contraction	1.06 ± 0.36
The number of cases which required catheter reposition	6 (23)
Cases with unilateral response	16 (62)
Success	13 (81)
Partial success	3 (19)
Failure	0 (0)
Cases with bilateral response	10 (38)
Success	8 (80)
Partial success	1 (10)
Failure	1 (10)

Values are either the mean ± SD or the number of patients (%). A total of 26 patients in the group E were confirmed the responses to electrical stimulation

The rate of “complete success” was significantly higher in the group E (53.6% for group L versus 80.8% for group E) (Table 3). In addition, the sensitivity of LOR + electrical stimulation in finding epidural space in the group E was 0.961 (25/26), whereas the sensitivity of LOR in the group L was 0.679 (19/28) (Table 3). Especially the number of “failure” of group L was much higher than that of group E while the numbers of “partial success” were comparable between the two groups (Table 3).

**Table 3:** Clinical outcomes

	Group L (***n*** = 28)	Group E (***n*** = 26)
Primary outcome (complete success / partial success / failure)	15 / 4 / 9 (54 / 14 / 32)	21 / 4 / 1^*^ (81 / 15 / 4)
Sensitivity in finding the epidural space	0.68	0.96^*^
Maternal satisfaction	3.39 ± 0.73	4.04 ± 0.72^*^
Ephedrine or phenylephrine use during operation / PACU	1.23 ± 1.46 / 0.32 ± 0.67	0.89 ± 1.07 / 0.23 ± 0.59
Hypotension (Y / N)	17 / 11 (61 / 39)	15 / 11 (58 / 42)
Nausea (Y / N)	2 / 26 (7 / 93)	1 / 25 (4 / 96)
Pain score (VAS) at discharge in the PACU	1.25 ± 1.40	1.65 ± 1.41
1 min Apgar	8.18 ± 1.16	7.69 ± 1.72
5 min Apgar	9.54 ± 0.58	9.23 ± 0.82

Values are either the mean ± SD or the number of patients (%). Group L used a loss of resistance technique in the epidural cesarean section while group E used electrical epidural stimulation combined with a loss of resistance technique. Sensitivity in finding the epidural space was calculated as “complete success + partial success / complete success + partial success + failure” in each group. The calculation of the specificity was not available due to the methodological limitation. Maternal satisfaction was rated on a scale ranging from 1 (*very unsatisfied*) to 5 (very *satisfied*). Group E showed higher success rate, sensitivity in finding the epidural space and maternal satisfaction compared to group L. The other outcomes were comparable between the two groups. ^*^p < 0.05 compared to group L. *Abbreviations*: *VAS* Visual analogue scale, *PACU* Post-anesthesia care unit

Maternal satisfaction of group E was significantly higher than that of group L (Table 3). Other secondary outcomes including Apgar scores, presence or absence of hypotension and ephedrine or phenylephrine use, anesthetic times, fluids, transfusion, urine output, blood loss, nausea, and pain score were comparable between the two groups (Tables 3, 4). Overall incidences of hypotension and severe nausea were 59% (32/54) and 5.6% (3/54), respectively (Table 3). No incidence of severe complication or catheter-related complication was reported.

**Table 4:** Required time and intake/output in the perioperative periods

	Group L (***n*** = 28)	Group E (***n*** = 26)
Time interval between the admission to the operating room and the start of the operation (min.)	32.37 ± 12.46	27.81 ± 15.92
Time A (interval between drape and test dose administration) (min.)	8.79 ± 3.97	8.81 ± 3.20
Time B (time it took to confirm epidural space, administer main dose and to place epidural catheter) (min.)	4.61 ± 2.50	4.67 ± 2.16
Operation time (min.)	48.93 ± 11.61	50.77 ± 16.82
Time interval between the end of the operation and the discharge to PACU (min.)	6.70 ± 4.11	6.42 ± 3.21
Crystalloid (ml)	1060.37 ± 469.41	1128.08 ± 388.15
Colloid (ml)	37.04 ± 133.44	53.85 ± 152.92
Transfusion (pint)	0.12 ± 0.43	0.08 ± 0.39
Urine output (ml)	249.82 ± 144.49	224.23 ± 115.27
Blood loss (ml)	479.63 ± 138.74	519.92 ± 209.76

Values are the mean ± SD. Group L used a loss of resistance technique in the epidural cesarean section, whereas group E used electrical epidural stimulation combined with a loss of resistance technique. Perioperative time period in each step and intake / output during perioperative periods were comparable between the two groups. *Abbreviation*: *PACU* Post-anesthesia care unit

## Discussion

In the present study, we showed the usefulness of electric-guided epidural confirmation with LOR technique. In addition to higher maternal satisfaction, there was a significant increase in success rates without increasing any complications.

Compared to epidural anesthesia, spinal anesthesia has several disadvantages including the risks of the extensive block, postdural puncture headache, and abrupt hypotension. Despite these shortcomings, the higher success rate of spinal anesthesia in comparison to that of epidural anesthesia (94% vs 76%) [4] may be the one of the important advantages. Conversely, if success rate of epidural anesthesia improves, the merit of spinal anesthesia diminishes.

After epidural anesthesia have been introduced in 1921, LOR technique and hanging drop technique were independently developed to identify the epidural space in 1932 [1]. And LOR technique is more commonly used especially in the lumbar spine compare to the hanging drop technique because there was a lack of evidence for intrinsic negative pressure in the lumbar epidural spaces [10]. However, the loss of resistance seemed to be more difficult to feel in the parturient. Lechner, et al. [11] reported lower maximum pressure just before loss of resistance and higher pressure in the epidural space in the parturient, compared to those in the non-parturient probably due to weakened ligament flavum and engorged epidural vein.

There were several other methods to identify the epidural space. C-arm guided epidural confirmation is not used in the obstetrics due to radiation exposure. Sonographic guided epidural confirmation can be considered as an option these days, and the use of ultrasonography is increasing with improvements in ultrasound technology [12]. However, ultrasonography requires relatively expensive equipment. Some other ways to confirm the epidural space include epidural waveform analysis [13] and electrical stimulation that we discuss in the current study.

Tsui et al. [14] first described the use of electrical stimulation to confirm catheter placement in the epidural space. Subsequently, several researchers applied epidural stimulation for catheter placement [15–17]. Previous studies showed favorable results without side effects. However, it was difficult to unify diverse guidelines. In particular, different catheter types required different reference ranges for electric current. Tsui’s method, where an epidural catheter was used with a fixed electrode at the distal tip and the electric impulse was conducted through normal saline within the lumen of catheter [14], required a relatively high electric current (1–10 mA at a pulse width of 0.3 ms) to produce motor response [18]. Similar to our catheter, Charghi et al. [19] used a single-port, metal coil-reinforced catheter containing a removable stylet for electric conduction instead of normal saline as a priming solution. Their results were comparable to ours, but the mean stimulatory current threshold was different from Tsui’s. Although the absolute value provided in Tsui’s criteria was not applicable in different settings, at least it can signal catheter misplacement of intrathecal or epiradicular spaces when the values of the required current needed for electrical response were too low [16, 17].

The success rates of epidural anesthesia are affected by the practitioner’s skill. A non-obstetric anesthesiologist’s care was regarded as one of the risk factors for epidural failure when provided not only in primary epidural anesthesia for cesarean section but also in conversion of labor epidural analgesia to cesarean delivery anesthesia [6]. There were several ways to define epidural failure, such as conversion to other forms of anesthesia [20], pain during surgery [21], and failure to achieve the predefined degree of nerve block [22]. We saw that failure is due to two reasons: a) failure in finding the epidural space at all and b) mismatched level or unilateralization of drug spreading out on the epidural space. In our study, the number of “failure” in the group E was significantly reduced compared to group L. This finding suggests that the electric confirmation was effective for untrained practitioners in confirming the epidural spaces.

We considered the level of anesthesia and drugs used for epidural anesthesia. To find an insertion point, the Tuffier’s line was used as a reference in our study. We regarded the transverse line connecting the tops of the iliac crests as the Tuffier’s line. Kim, et al. [23] showed that the mean vertebral level of Tuffier’s line in the parturient women was L3-lower vertebral level which was higher than that of non-parturient women (L4-lower vertebral level). Therefore, our target level (L2/3) of epidural injection was the level above the Tuffier’s line. However, some cases that deviate from the average in the actual vertebral level were reported [23], so knowing the correct level in advance may improve the results regarding “partial success”. Next, there were several concerns about the usefulness of epidural test dose in obstetrics [24]. Local anesthetics may cause unwanted prolonged motor block, but it was not a major problem in the cesarean section. However, epinephrine was not recommended due to its restricted sensitivity [25] and risk exposures regarding high myocardial oxygen consumption and low uterine blood flow [24]. For these reasons, we used lidocaine only as a test dose to exclude an intrathecal injection. We also considered lidocaine as main dose for its fast onset and ropivacaine as supplementary dose for anesthetic quality [26]. Because of the simplicity of our regimen (no mixed solution), the risk of drug misadministration by novices appeared to be minimal, and once the epidural has been found, electrical stimulation did not seem to have a significant effect on the partial success rate.

The other outcomes in the study showed the efficiency and the safety of electrical stimulation. In almost all cases, electrical stimulation was performed during the wait time after the test dose administration. Although 6 patients in the group E required catheter repositioning due to lack of response to the electrical stimulation, the delay time was up to 5 min from the average. The difference in Time B, which includes the time for catheter placement, was only 0.06 min between the two groups (Table 4). Therefore, the risk of time consumption was minimized. During the study, no catheter-related adverse event including intrathecal or intravascular injection was reported, and the electrical stimulation did not affect the Apgar score. The most common complication was hypotension, which appeared to be a side effect from the neuraxial block. In addition, the incidence of hypotension and the use of cardiovascular drugs during perioperative period were comparable between the two groups. Most of the hypotensive events were gradually developed, and all were successfully resolved without any sequelae.

Before conducting this study, we tried to stimulate the exact level by identifying the corresponding radiating paresthesia, but the electrical stimulation seemed to have a low spatial resolution. In addition, when the catheter was located to one side, the nerve roots were easily stimulated, and the current value was lowered. There were also individual variations and a wide range of required currents due to possibility of epidural adhesion, epidural vein engorgement or any other anatomical variations. For these reasons, electrical stimulation has a limitation in confirming an appropriate level of anesthesia. Fortunately, most of the unilateral stimulations resulted in bilateral anesthesia. Large doses of local anesthetics seemed to extend to the contralateral side. Therefore, as a straightforward guideline, once a reliable response to the stimulation is detected even in unilateral side, we recommend proceeding to the next step.

There were other limitations as well. First, the usefulness of electrical stimulation may not be evident for the well-trained anesthesiologist. In statistical perspective, the higher success rate in the LOR-only group requires the greater number of sample size to have a significant meaning. Second, the guidewire inside the epidural catheter may increase a risk of incidental intrathecal or vascular puncture in theory. However, this issue has not been reported in this study as well as in Charghis’s study [19] which was similar to ours. Third, there was a methodological limitation in allocation concealment (double blind). During the electrical stimulation, the patient and the practitioner may notice the group assignment. However, another blinded independent anesthesiologist assessed the outcomes in the postoperative period to minimize the performance bias. Finally, the use of a test dose could require more electrical current to overcome sensorimotor blockade of lidocaine although the test dose did not appear to have a significant effect in this study.

Despite these limitations, our study suggests several advantages and implications. First was the type of catheter. There was a risk of air lock which disturbs the current flow down the column of saline contained inside the Tsui’s catheter [14]. In our study, we used the epidural catheter with a built-in conductive guidewire. Our devices seemed to be simple while reducing the risk described above. The minimum currents of our device were also lower than other electrical epidural stimulation methods. This may reduce the risk of injury from electrical stimulation. Second, the method and the results in the present study may contribute as a guideline and reference values when using our catheter. However, it should be noted that the reference value can be changed in other situations including non-obstetric patients or patients known to have history of lumbar disease or surgery. Third, high maternal satisfaction suggested that electrical stimulation did not appear to be an unpleasant or uncomfortable experience to the patient. In fact, no complaints or problems with electrical stimulation occurred during the study. To our knowledge, this study was the first application of epidural electrical stimulation for cesarean section. Although the method we improved had not changed dramatically, the impact has been substantial. Taken together, at least in the obstetric area, our findings supported the usefulness of electrical stimulation.

We think that our method would be more improved when used in conjunction with ultrasonography because ultrasound guided technique may provide a desired level of anesthesia. In detail, we recommend planning the epidural technique in advance while using ultrasonography to determine the level of vertebral body and the depth and location of epidural space, and then confirming with electrical stimulation. Further studies are required to determine whether the combined use of electrical stimulation and ultrasonography is useful for difficult epidural procedures.

## Conclusion

Confirmation of the epidural space using electrical stimulation improved success rate as well as maternal satisfaction without wasting time, fetal distress or any other risks in the cesarean section. It seemed to be effective for inexperienced clinicians and those with recent gap in experience.

## Data Availability

The datasets of the current study are available from the corresponding author on reasonable request.

## Abbreviations

LOR: Loss of resistance
PACU: Post-anesthesia care unit
VAS: Visual analogue scale
